# In Vivo Evaluation of Regenerative Osteogenic Potential Using a Human Demineralized Dentin Matrix for Dental Application

**DOI:** 10.3390/dj12030076

**Published:** 2024-03-18

**Authors:** Nessma Sultan, Soher Nagi Jayash

**Affiliations:** 1 Oral Biology, Faculty of Dentistry, Mansoura University, Mansoura 35516, Egypt; nesmasultan88@gmail.com; 2 Oral Biology and Dental Morphology, Faculty of Dentistry, Mansoura National University, Gamasa 7731168, Egypt; 3 Roslin Institute, University of Edinburg, Edinburgh EH25 9RG, UK

**Keywords:** demineralized dentin matrix hydrogel (DDMH), nano-hydroxyapatite hydrogel (NHH), hydrogels, bone regeneration

## Abstract

Background: The use of a demineralized dentin matrix (DDM) has garnered substantial importance in dentistry. This study was carried out to evaluate the osteoinductive performance of DDM in comparison to nano-hydroxyapatite (n-HA) on calvarial critical-sized bone defect. Methods: Two critical-sized defects (CSDs) were bilaterally trephined in the calvarium of sixteen healthy white rabbits. The rabbits were categorized into four groups: in group 1, the defect was left empty; in group 2, defects were filled with sodium alginate (SA) hydrogel as a sole material; in group 3, defects were treated with nano-hydroxyapatite hydrogel (NHH); in group 4, defects were treated using demineralized dentin matrix hydrogel (DDMH). Histological and immunohistochemical analyses were carried out to evaluate the total areas of newly formed bone. Results: The DDMH group showed that new woven bone tissue progressively bridged the defect area while there was no bone in the control group. Collagen expression was significantly different in the DDMH- and NHH-treated groups compared to in the SA group at 4 and 8 weeks (*p* < 0.01). OCN expression was significantly higher in the DDMH group in comparison to in the NHH or SA groups at 8 weeks (*p* < 0.01). Conclusions: The DDMH group exhibited significantly higher levels of new bone formation compared to the NHH group at both 4 and 8 weeks post-surgically.

## 1. Introduction

Annually, a proportion ranging from 3% to 5% of the global population undergoes medical implant procedures. Among these procedures, approximately 50% are attributed to the replacement of deteriorated joints and the mending of fractured bones. However, repairing bony damage encountered by trauma or developmental disorders affecting craniofacial structures is considered a permanent problem for regenerative procedures [1,2,3]. Consequently, research into new or improved materials for bone regeneration became crucial to decrease the cost of the materials that are going to be used in bone regeneration.

Autologous bone grafts are frequently utilized to repair bony defects within the cancer resection or after severe trauma. The utilization of biological materials in regenerative medicine has witnessed significant advancements in recent years. Among these materials, the use of a human demineralized dentin matrix (DDM) has emerged as a promising candidate for bone regeneration due to its inherent osteoinductive properties. DDM possesses a complex composition of growth factors, proteins, and extracellular matrix components, making it an ideal substrate for stimulating osteogenesis [4,5].

Dentin, comprising 70% inorganic and 30% organic components, mirrors the composition of alveolar bone (65% inorganic and 25% organic components). Dentin’s inorganic fraction primarily comprises hydroxyapatite, while its principal organic content, type 1 collagen, plays an important role in both bone regeneration and mineralization processes. This unique composition underscores its significance in bone regeneration and highlights its potential as a valuable therapeutic agent [6].

Hydrogels, three-dimensional networks of hydrophilic polymers, have garnered immense interest in the biomedical field owing to their excellent biocompatibility and tunable properties. Different natural polymers, including collagen, sodium alginate (SA), chitosan and hyaluronic acid can be used to make natural-based hydrogels. Combining the osteogenic potential of DDM with the versatility of hydrogels offers a compelling avenue for creating advanced scaffolds that can support bone regeneration in vivo. This synergy between DDM and hydrogels represents a novel approach in the field of regenerative medicine, with the potential to address critical challenges in bone tissue engineering [7,8,9]. A previous publication highlighted the osteoinductive role of SA hydrogel loaded with DDM in vitro [10], thus this study aims to evaluate the osteoinductive capability of SA hydrogel loaded with either DDM or n-HA in a rabbit calvarial defect by histological and immunohistochemical analyses. This study explores the practical application of human DDM in regenerative dentistry. It delves into the assessment of its regenerative potential in vivo, specifically focusing on its ability to promote osteogenesis, which is crucial for dental procedures requiring tissue regeneration. This study investigates the efficacy and suitability of human DDM as a viable option for enhancing dental regeneration, offering insights into its potential implications for dental treatments and procedures.

## 2. Materials and Methods

A sample of ten (*n* = 10) caries-free permanent human premolar teeth, which were extracted for orthodontic purposes, were obtained from the outpatient clinic in faculty of dentistry, Mansoura university, Egypt. The surgical procedures were conducted at Mansoura university experimental research center. The university ethical committee granted approval for the collection and preservation of teeth, animal handling, and all experimental protocols under the reference number A23060722.

### 2.1. Preparation of Dentin Particles

Based on the previously published studies [10,11,12], the teeth dentin was ground followed by partial demineralization within a declining titer of ethylenediamine tetra acetic acid (EDTA) concentrations as follows: 17% (5 min), 10% (5 min), and 5% (10 min). Then, the teeth particles were ultrasonically cleaned while embedded in distilled water for a duration of 10 min followed by freeze-drying. 

### 2.2. Preparation of SA Solution

A concentration of 5% (*w*/*v*) of SA (Sigma-Aldrich, St. Louis, MO, USA) was dispersed in distilled water and glycerol was used as a plasticizer. Then, the filtration of this solution was performed using 0.45 μm syringe filters (Thermofisher scientific, Altrincham, UK). 

### 2.3. Preparation of SA Hydrogel, DDMH and NHH

DDM particles were dispersed in the SA solution to reach a mass ratio of 1:1 [12], while n-HA powder (particle size less than 200 nm, purchased from Sigma-aldrich, St. Louis, MO, USA) was combined with SA solution in a final concentration of 5 mg/mL [13]. The hydrogels were formed by addition of a sterile solution of CaCl_2_ 5% (*w*/*v*) during homogenization of the gel at 10,000 rpm. At the end of this process, the gels were homogenized for 2 cycles at 10,000 rpm for 5 min to obtain the injectable form of either SA hydrogel, DDMH and NHH.

### 2.4. Characterization of the Formulated Hydrogels

A dynamic light scattering device (Zeta sizer Nano ZS90, Malvern, Worcestershire, UK) was used to measure the zeta potentials of n-HA and DDM particles as well as their particle sizes. The measurements were taken at a fixed angle of 173° at 25 °C. Prior to analysis, all of the samples were diluted using distilled water. All characterization was conducted before, in our previously published manuscript [10]. 

### 2.5. Experimental Animals 

#### 2.5.1. Sample Size Calculation

Sixteen male rabbits of the New Zealand white breed, deemed to be in good health, were selected for this study. In each group, four rabbits were used; two rabbits were sacrified after 4 weeks, and the other two rabbits were scarified after 8 weeks. The group size was based on power analyses informed by our published data (histological analysis). We aimed for a significance level of 5% and a power of 95%. From prior experience, 4 defects (2 animals) per group are normally sufficient for this experiment [8].

The rabbits had an average weight ranging from 1.5 to 2 kg. Female rabbits were excluded from this study as hormonal changes may affect the results. The rabbits were individually housed in separate cages, adhering to the optimal experimental settings as outlined by the animal ethics committee rules. During the trial time, the animals were provided with a regular pellet meal and tap water for their sustenance. The ambient temperature within the chamber was between 22 and 24 degrees Celsius, while the animals were subjected to a light/dark cycle of 12 h of light followed by 12 h of darkness.

#### 2.5.2. Surgery of Experimental Animals 

**Figure 1:**
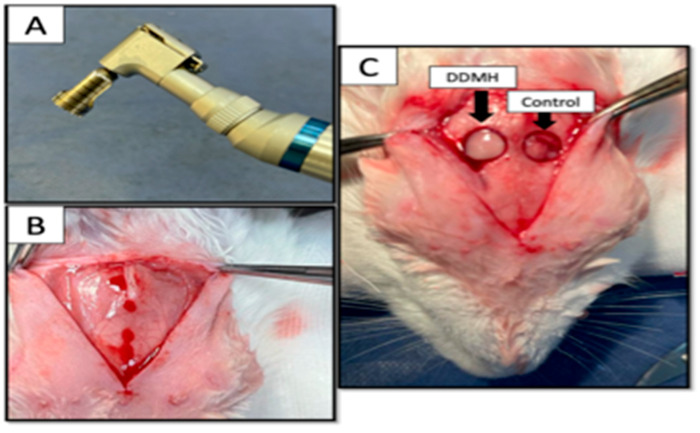
Photographs showing (**A**) the trephine bur used in the cavity preparation, and (**B**) exposing the calvarium; (**C**) CSDs (8 mm in diameter) were trephined bilaterally within the calvarial bone. DDMH was applied in the right defect as an example of application, while the left remained empty as an example of the control group.

Every rabbit was administered an intramuscular injection of Zoletil 50 (0.5 mL/kg) (Virvac, Nice, France) and Xilazine (0.25 mg/kg) (Rompum, Bayer, Leverkusen, Germany) to induce general anesthesia. A calvarium full-thickness defect was made in the experimental animals. Briefly, a sagittal midline cranial incision was made to expose the calvarium with the sterilized #15 scalpel blade. Using a low-speed contra-angle handpiece (NSK, latch bur, Tokyo, Japan) with a 8 mm trephine bur with an inner diameter of 8 mm and an outer diameter of 9 mm (3i, Palm Beach Gardens, FL, USA) (Figure 1). The 8 mm defects were created bilaterally: 2 mm laterally to the midline. Without damaging the dura mater, the circular bone fragment was then gently removed with a periosteal elevator. 

#### 2.5.3. Postoperative Care 

Using resorbable 3-0 Vicryl sutures (Ethicon^®^, Johnson & Johnson, Somerville, NJ, USA), the overlying soft tissues and skin were stitched in layers. The incision site was cleaned with betadine antiseptic solution using sterile gauze. Each experimental animal received antibiotics (cefotaxime 1 g vial Egyptian International Pharmaceutical Industries, Ramadan, Egypt) every 24 h and analgesics (Voltaren 75 mg/3 mL Novartis, Giza, Egypt) for 2 days postoperatively. Animals were sacrificed at 4 and 8 weeks post-surgically. Then, the calvaria bones containing the healed sites were surgically harvested and immediately fixed in neutral-buffered formalin (10%) for 24 h.

#### 2.5.4. Histological Analysis

For histological analysis, four rabbits were sacrificed for each group, two rabbits were scarified per time point. After the skulls were removed, a hard tissue cutting machine was used to harvest the area of interest. Following the appropriate 10% neutral formalin fixation of the harvested tissue slices, the proteins inside the tissue were preserved. After this, the tissues underwent decalcification by immersion in a 10% EDTA solution (pH 7.2): the calvarium samples typically required 3–4 months to be ready for tissue processing. Subsequently, the samples were embedded in paraffin; and after staining with hematoxylin and eosin (H&E), Masson’s trichrome (MT) (Cat# HT15, Sigma Aldrich), and immunohistochemical staining with a polyclonal anti-rabbit osteocalcin antibody (OCN; Cat# GB11233, diagnostic biosystems) at a 1:150 dilution, histological examination was conducted using an Olympus BX-51 optical microscope (Olympus Co., Tokyo, Japan).

#### 2.5.5. Immunohistochemical Staining 

Using a conventional streptavidin-biotin immunoperoxidase approach, osteocalcin antibody (OCN; Cat# GB11233, diagnostic biosystems) was used for the immunohistochemical examination. After the sample was fixed in paraffin, histological slices measuring 3 µm in thickness were extracted, deparaffinized, and hydrated. The formalin pigments were removed with a 10 min soak in a solution containing 10% ammonium hydroxide and 95% ethanol, which was followed by a washing in running and distilled water. The tissue slices were treated with 1% pepsin solution (pH = 1.8) at 37 °C for 60 min to extract the antigen from the tissue. After that, the samples were cleaned and blocked with 3% hydrogen peroxide. The solution was then removed by washing the specimens under running water and then changing them twice with distilled water. The cuts were quickly incubated at pH 7.4 in PBS solution (BSA; Biotest S/A, São Paulo, SP, Brazil) so as not to drastically change the ph. The main antibody, polyclonal osteocalcin, was diluted 1:150 in PBS solution with 1% bovine albumin and 0.1% sodium azide, and the slides were incubated overnight at 4 °C in a damp chamber. The primary antibody was not incubated; instead, the identical slices served as a negative control. The specimens were subjected to an incubation period of 3 min in a dark chamber, with 300 mg of diaminobenzidine as chromogen (3,3-diaminobenzidina; Sigma Chemical CO., St. Louis, MO, USA) in 100 mL of PBS solution, pH 7.4. After 3 min of hematoxylin counterstaining, the slides were cleaned. The slides were dehydrated using an increasing ethanol series, cleaned in xylene, and then placed on Permount^®^ (Fischer Scientific, Fair Lawn, NJ, USA) for light microscopy examination. 

#### 2.5.6. Image Analysis

Four tissue slides were prepared for control and experimental groups at each time point. Four randomly selected regions in the middle of the defect site were captured at a magnification of 100×. Subsequently, 16 images per group per time point were utilized for image analysis, the ratio of newly formed bone to the entire defect area in the slide images was calculated. This analysis was performed utilizing the Intel^®^ Core I7^®^ based computer and VideoTest Morphology^®^ software (Version 5.0, Russia), which features a specific built-in routine for area and percentage area measurements. The images used for analysis were stained with MT and OCN. In MT staining, mature bone was indicated by red, whilst regenerated bone, collagen fibers, or osteoid were indicated by blue. In OCN immune staining, the positive reaction appeared in brown color.

#### 2.5.7. Study Design and Grouping 

Two defects were prepared in each animal and were filled with the same type of each of the tested materials. This study comprised four distinct groups based on the treatments administered to cover the defects: In group 1, designated as the control group, manual pressure hemostasis was applied using gauze without any additional therapy in the circular defect. In contrast, group 2 addressed calvarial bone defects by exclusively utilizing SA hydrogel as the grafting medium. Group 3 employed the application of NHH to treat bone defects. In group 4, DDMH was utilized for the same purpose. A volume of 50 µL of the tested hydrogels was added to each circular defect (Figure 1C). 

#### 2.5.8. Statistical Analysis

Defects were utilized as statistical units; two defects were prepared in each animal so a total of eight statistical units were assigned to each group at two different time points (*n* = 8). Data are presented as the means ± SEM. Data analysis was performed using Prism (version 3.1) with one-way ANOVA and Tukey’s post hoc test. Specific test details are provided in the legends of each figure. Significance levels were set at * *p* < 0.05, ** *p* < 0.01, *** *p* < 0.001, and **** *p* < 0.0001. 

## 3. Results

All the animals used in this study were survived without any complications. At the 8th week, the defect sites showed no indications of necrosis, hematoma, or infection. Under all bone defect sites, the dura mater and brain tissues showed no clinical signs of inflammation, creation of scars, or unfavorable tissue reactivity to the grafting materials. After the procedure, all of the rabbits were able to feed themselves and resume their regular stride after recovering from anesthesia; and during the healing process, they displayed no symptoms of discomfort or evidence of infection, like a red, hot incision or exudate.

### 3.1. Histological Results

Given that cavities were meticulously trephined bilaterally in 16 rabbits, a total of 32 critical-sized defects (CSDs) were made available for subsequent microscopic analysis. The distinct contrast between the pre-existing lamellar and less cellular calvarial bone, juxtaposed with the freshly created bone characterized by numerous osteocytes, facilitated an easy and clear identification along the borders of the CSDs. Slicing the calvarial bone into cross-sections and applying stains such as H&E, MT, and OCN enabled a thorough analysis of the regeneration pattern and provided confirmation of successful bone regeneration.

#### 3.1.1. H&E Results

##### After 4 Weeks

**Figure 2:**
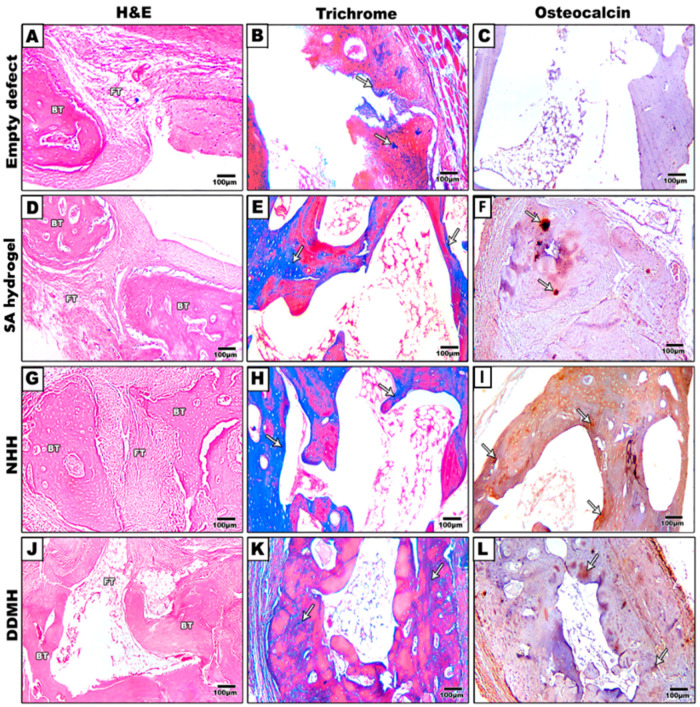
Representative histological images of (H&E) (**A**,**D**,**G**,**J**), Masson’s trichrome (**B**,**E**,**H**,**K**) and osteocalcin immune staining (**C**,**F**,**I**,**L**) are shown at 4 weeks post-treatment. Coronal sections through the mid-point of the defects were prepared from decalcified specimens. Fibrous connective tissues were seen to be mostly filled in the defect areas in the control group (**A**–**C**). The group treated with SA hydrogel as the only grafted material showing fibrous and adipose tissue (**D**–**F**). lacuna spaces were observed in the newly formed lamellar bone in NHH with a wide bone marrow space (**G**–**I**). DDMH-treated groups showed considerable new bone trabeculae formation (**J**–**L**). BT: bone trabeculae; FT: fibrous tissue. Arrows represent MT- and OCN-positive reactions. Scale bar: 100 μm.

**Figure 3:**
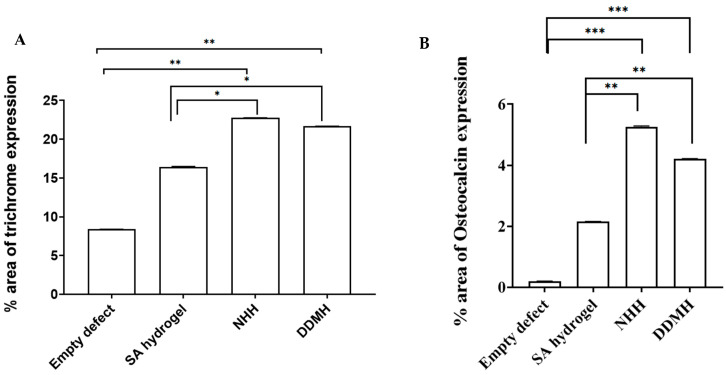
The histomorphometric analysis of MT- (**A**) and OCN- (**B**) positive expressions at 4 weeks after treatment. Data are expressed as the means ± SEM. * *p* < 0.05 and ** *p* < 0.01 and *** *p* < 0.001 indicate significantly differences.

The control group displayed an irregular, thin layer of fibrous connective tissue filling the defect, devoid of any new bone formation. In the SA-treated group, osteoblasts were observed lining the bone trabeculae amidst predominantly fibrous connective tissues. The defect exhibited partially aligned connective tissues, with limited new bone formation at the margin. The NHH group demonstrated considerably more woven bone development at the defect edge compared to the SA-treated group. Notably, numerous osteocytes were present within the bone trabeculae, indicating an ongoing active bone formation process. In the DDMH group, the development of woven bone tissue progressively bridged the defect area, suggesting a gradual and promising bone advancement (Figure 2 and Figure 3).

##### After 8 Weeks

**Figure 4:**
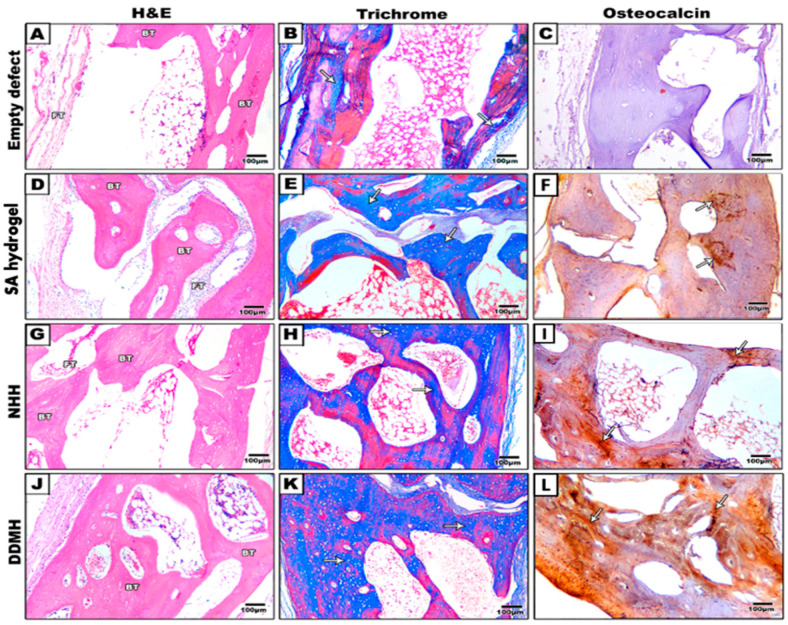
Representative histological images of (H&E) (**A**,**D**,**G**,**J**), Masson’s trichrome (**B**,**E**,**H**,**K**) and osteocalcin immune staining (**C**,**F**,**I**,**L**) are shown at 8 weeks post-treatment. Rather than the newly created bone, the defect locations in the control group were found to be primarily filled with fibrous connective tissues and adipose tissue (**A**–**C**). Circular new bony islands with the presence of early woven bone were observed in the group treated with SA as the only grafted material (**D**–**F**). Newly formed lamellar bone was observed in NHH with a wide bone marrow space (**G**–**I**). In contrast, DDMH-treated groups showed considerable new bone formation at 8 weeks with relatively narrow marrow spaces (**J**–**L**). BT means bone trabeculae; FT: fibrous tissue. Arrows represent MT- and OCN-positive reactions. Scale bar: 100 μm.

**Figure 5:**
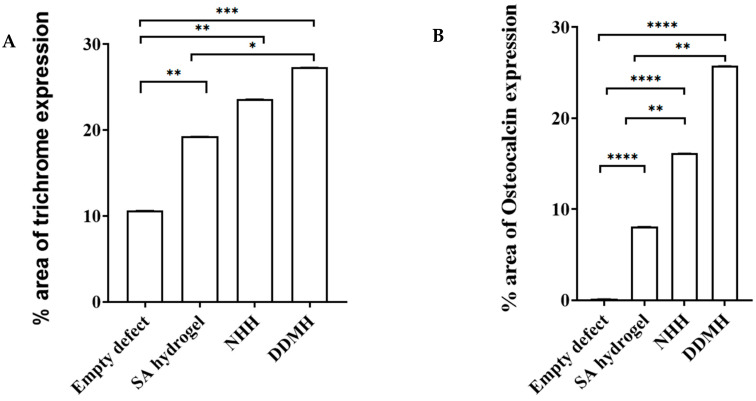
The histomorphometric analysis of MT- (**A**) and OCN- (**B**) positive expressions at 8 weeks after treatment. Data are expressed as the means ± SEM. * *p* < 0.05 and ** *p* < 0.01, *** *p* < 0.001, and **** *p* < 0.0001 indicate significantly differences.

In the control group, the defect was filled with fatty tissue and fine fibrous connective tissue. Group 2, treated with SA hydrogel, exhibited uniformly dense and well-aligned connective tissue within the defect, accompanied by observable newly formed bone. The NHH group displayed woven bone formation bridging the defect margin, progressively undergoing calcification. While evidence of new bone growth was present, signs of immature bone formation, including a sizable medullary cavity, were also noted. In the DDMH group, newly formed bone tissue showed continuous and progressive development, completely bridging the defect area, not just along the fracture margin but throughout the entire defect space. A substantial amount of new bone was observed, covering the central region of the defect. Compared to the NHH group, there was greater new bone growth, although the medullary cavity appeared smaller in size (Figure 4 and Figure 5).

As depicted in Figure 2 and Figure 4, the calvarial bone in the control group showed no signs of repair even after 8 weeks post-surgery. Conversely, in the three groups that received hydrogel treatments, the gap gradually closed over time. In the NHH and DDMH groups, lacunae formed, and osteocytes were visible after 4 weeks, indicating an initial stage of bone formation. However, by the 8 week mark, the trabecular bone had thickened, the marrow had integrated, and clear lamination was evident, signifying a more matured bone structure.

#### 3.1.2. Masson Trichrome Staining Results 

To assess bone maturity, MT staining was conducted.

##### After 4 Weeks

Within the calvarial defects, the blue coloration indicative of collagen deposition was notably visible, particularly accentuated in the three hydrogel-treated groups compared to the control group. This contrast strongly implied that bone matrix synthesis was significantly less active in the control group at 4 weeks post-surgery. Conversely, in the DDMH, NHH, and SA groups, collagen deposits were prominently present at 4 weeks, indicating an early and active stage of bone matrix synthesis, with percentages of 21.67% ± 0.016, 22.74% ± 0.033, and 16.40% ± 0.00, respectively. Image analysis further revealed no significant difference in collagen expression between the DDMH- and NHH-treated groups 4 weeks post-surgery, although both exhibited a notable difference compared to the SA group (Figure 2 and Figure 3A). 

##### After 8 Weeks 

The newly formed bone became notably more prominent (DDMH: 27.29% ± 0.012, NHH: 23.57% ± 0.023, SA: 19.25% ± 0.003) being significantly higher in the DDMH group in comparison to the NHH and SA groups. This probably suggests a significant progress in new bone formation, particularly in the DDMH group, compared to in the NHH and SA groups. Additionally, a significant difference in collagen expression was observed between the NHH and SA groups. Note that bone maturation was still not obvious, suggesting that extra time may be needed to achieve complete bone maturation (Figure 4 and Figure 5A).

#### 3.1.3. Immunohistochemical Staining

To quantify bone regeneration degrees, the newly formed bone in calvarium was assessed using OCN staining calculating the percentage of brown positive expression revealed the following results: in the control group, the expression was 0.20% ± 0.001 at 4 weeks and 0.141% ± 0.001 at 8 weeks. For the SA group, the expression was 2.15% ± 0.005 at 4 weeks and 8.10% ± 0.002 at 8 weeks. In the NHH group, the expression was 5.25% ± 0.001 at 4 weeks and 16.17% ± 0.002 at 8 weeks. In the DDMH group, the expression was 4.21% ± 0.004 at 4 weeks and 25.76% ± 0.008 at 8 weeks (Figure 3B and Figure 5B). These findings suggest varying degrees of bone regeneration efficacy among different treatment groups over the 4- to 8-week period, with DDMH showing the most promising outcomes.

The increase in OCN expression markedly increased in the DDMH group from 4 to 8 weeks. Note that after 4 weeks, there was no significant difference in OCN expression among the DDMH and NHH groups, while after 8 weeks of treatment, the brown positive expression of OCN was significantly higher in the DDMH group in comparison to in the NHH or SA groups. Also, the results revealed that there was no statistically significant difference between the SA group and the NHH group at 4 weeks post-surgically (Figure 3B and Figure 5B). These findings suggest that DDMH treatment may have a substantial impact on bone repair and maturation, particularly evident in the extended 8-week period, while differences between other treatment groups may become more pronounced over time.

## 4. Discussion

The originality of the topic lies in its exploration of a novel therapeutic avenue in regenerative dentistry, its focus on evaluating the osteogenic potential of a human dentin matrix in vivo, and its implications for enhancing clinical outcomes and shaping future research endeavors in the field. Histomorphometric analysis results in our current comparative investigation of bone healing and osteogenesis in rabbit calvarial defects showed that, after 8 weeks, DDMH had a better ability to form new bone than SA hydrogel alone, indicating a possible osteogenic role for DDM. 

The rabbit model of calvarial defect is known to be helpful for analyzing and assessing the osseus formation potential of grafting materials. The calvarial bone has the benefit of being biomechanically similar to the jaw bones of humans since it is made up of trabecular bone in between the inner and outer cortical bones [14,15]. Male rabbits were the only experimental subjects since female rabbits’ hormonal changes or pregnancy could alter the outcomes of the experiment. Experiments were performed under identical settings. The CSDs in rabbit calvarium were 8 mm in diameter, which is considered a small defect in an animal with no more than 10% bone regrowth and will not heal spontaneously. The rabbit’s cranial dura mater can be seen whenever resected bone was removed after a trephine bur creates a circular bone defect having a diameter of 8 mm with full depth. Because the cranial dura mater is crucial to the bone development of bone graft materials, care was taken to protect it in these situations [16]. This study investigated bone repair in rabbits over two postoperative periods, 4 and 8 weeks, focusing on osteocalcin immune expression to track maturation. The extended 8-week period accounts for rabbits’ faster metabolism, potentially leading to improved outcomes. Previous research suggests that rabbit calvarial defects exhibit partial healing at 4 weeks and complete healing at 12 weeks post-surgery, prompting the authors to monitor bone formation before the 12-week mark.

Despite the fact that numerous studies have been carried out on rabbit calvarium to assess the osteogenic potential of various grafting materials [17,18,19], few studies have examined the possible effects of DDM as a bone grafting material [14,20]. After dentin demineralization, dentinal tubules become wider and serve as a channel for releasing essential proteins [21,22]. DDM is recognized to contain a variety of bone development factors, including bone morphogenic proteins and collagen-I, which are released following demineralization and are retained in dentin, demonstrating osteoinductive properties [14]. To the best of our knowledge, this is the first study to assess the osteogenic potential of DDM in vivo using it in a hydrogel form.

Injectable hydrogels resemble soft tissues and offer a medically appropriate environment for cell proliferation, which is why they are the preferred choice for bone repair. The hydrogels promote the physiological processes of cells by imitating the ECM’s high water content, adjustable porosity, and mechanical and physicochemical characteristics [23]. When it comes to porosity, toughness, and biological compatibility, SA-based hydrogels are highly advantageous and are frequently utilized as drug carriers in medical applications. For example, SA-based hydrogels may imitate the cartilage environment, encourage bone development, and aid in the healing of rat skull injuries [24,25]. For this reason, this study aimed to test the osteoinductive potential of SA hydrogel as a bone grafting material either alone or after being combined with DDM or n-HA. 

In this study, woven bone tissue was produced in the three hydrogel-treated groups, SA, DDMH and NHH, compared to in the control group because hydrogels arranged communication within defects and the original tissue offered an artificial environment for enhancing cell proliferation and differentiation. In a previous investigation, it was found that DDMH could promote bone formation in vitro, which is why we tried to test and further confirm the osteogenic potential of DDMH in vivo [10]. 

In the groups treated with DDMH, osteoblasts were gradually formed, and woven bone tissue was observed with interconnected bone trabeculae and relatively narrow marrow spaces, whereas in the NHH group, woven bone trabeculae was formed showing relatively wide marrow space at 8 weeks post-surgically. The histological analysis demonstrated that the defect was still visible in the control group and there were clearly fat and fibrous tissue present. Additionally, after 8 weeks, the restored bone in the control group had a worse quality due to the presence of fat and adipose tissue, suggesting impaired bone formation [26]. 

At the same time, osteoinduction occurred in the group treated with DDMH at 8 weeks post-surgically, significantly higher than what was seen in NHH group as demonstrated by OCN immune expression. Both the DDMH group and the NHH group reported that the defect’s periphery directly produced new bone. The NHH group showed a statistically significant higher level of new bone growth in bone defects at 4 or 8 weeks postoperatively compared to the SA group. Furthermore, at 8 weeks following surgery, the histological study showed that the DDMH group had substantially more new bone growth in the calvarial defect area than the NHH group. A similar finding was reported by Kim et al., who found significant volumes of new bone growth in the calvarium defects treated with DDM [14]. Note that Garca-Garca et al. came to the conclusion that SA hydrogel promotes bone formation in the CSD [26], which was similar to our finding, where an isolated bone trabeculae could be seen in the defect center of the SA hydrogel-treated group. 

Previous studies tried to test the osteoinductive potential of an un-demineralized dentin matrix on bone formation [27]. However, using a rat model, Mordenfeld et al. assessed how demineralization of dentin particles affected the growth of bone in an osteoconductive environment. Their research showed that when the degree of pre-grafting dentin demineralization increased, the rate of resorption of the dentin grafts increased [28]. One notable observation was the discovery that augmenting the extent of demineralization led to a substantial rise in bone formation, as evidenced by the presence of a low inflammatory response surrounding the dentin graft, suggesting that dentin can be integrated into bone tissue allowing for slow resorption and subsequent replacement with bone [29,30,31]. 

The primary inorganic component of teeth and bones is n-HA. Many studies have been conducted to investigate the usage of synthetic n-HA as a bone substitute due to its strong chemical resemblance to natural bone [32,33]. Since they may directly aid in tissue regeneration, cell adhesion and differentiation are crucial evaluation factors for tissue repair and regeneration [34,35]. Du et al. found that n-HA’s ability to promote cell adhesion was dubious with impaired cell proliferation on the surface of n-HA [36], although Liu et al. found that DDM might stimulate dental pulp stem cell adhesion, proliferation, and differentiation [37]. This could demonstrate how bone development differs in DDMH and NHH in our experiment.

The use of human DDM for regenerative purposes represents a novel therapeutic approach in dentistry. Unlike traditional methods, such as synthetic materials or autologous grafts, the utilization of a human dentin matrix offers a unique alternative with potential advantages in terms of biocompatibility, osteoinductivity, and availability. Its inherent biological compatibility with the recipient’s tissues minimizes the risk of immune rejection or adverse reactions. This characteristic enhances its appeal as a regenerative material for dental applications, potentially reducing complications associated with graft materials of non-human origin. This study’s focus on evaluating the osteogenic potential of human DDM sets it apart from previous research endeavors. By conducting in vivo assessments, the manuscript sheds light on the material’s ability to stimulate bone formation and regeneration within the dental context. This aspect underscores its clinical relevance and potential implications for enhancing the success rates of various dental procedures, such as bone augmentation and periodontal regeneration. Understanding the regenerative capabilities of a human dentin matrix in vivo provides valuable insights for clinicians and researchers alike, paving the way for the development of innovative treatment modalities and protocols aimed at improving patient outcomes in dental practice. While the current study contributes valuable insights into the regenerative potential of a human dentin matrix, it also highlights the need for continued research to optimize its clinical application, address potential limitations, and explore synergistic approaches that harness its regenerative properties more effectively.

In summary, the utilization of human DDMH emerges as a promising avenue for bone regeneration. Our study demonstrated the remarkable potential of DDMH in promoting new bone formation, bridging defects, and accelerating the healing process. Notably, DDMH outperformed other treatments, such as NHH and SA, showcasing its superior efficacy. This innovative approach holds great promise in the field of regenerative medicine, offering a valuable solution for enhancing bone regeneration and addressing critical challenges in orthopedic and dental procedures. As we move forward, further research and clinical trials are essential to fully unlock the therapeutic potential of DDMH and bring this advanced technique to practical applications, ultimately improving the quality of life of patients with bone-related disorders.

## 5. Conclusions

The findings indicate that the incorporation of DDM into SA-hydrogel enhanced the process of osteogenesis in rabbit CSDs. The significance of this becomes apparent within the clinical setting, particularly in the utilization of bone replacement materials. As we delve deeper into the osteogenic potential of DDM, we pave the way for innovative and effective therapies that can significantly enhance the quality of life of patients suffering from bone-related disorders.

## Data Availability

Data are contained within this article.

## References

[1] Kaito T., Hosono N., Makino T., Kaneko N., Namekata M., Fuji T. (2009). Postoperative displacement of hydroxyapatite spacers implanted during double-door laminoplasty. J. Neurosurg. Spine.

[2] Korkusuz F., Timuçin M., Korkusuz P. (2014). Nanocrystalline apatite-based biomaterials and stem cells in orthopaedics. Advances in Calcium Phosphate Biomaterials.

[3] Ferreira A.M., Gentile P., Chiono V., Ciardelli G. (2012). Collagen for bone tissue regeneration. Acta Biomater..

[4] Zizzari V.L., Zara S., Tetè G., Vinci R., Gherlone E., Cataldi A. (2016). Biologic and clinical aspects of integration of different bone substitutes in oral surgery: A literature review. Oral Surg. Oral Med. Oral Pathol. Oral Radiol..

[5] Agrali O.B., Yildirim S., Ozener H.O., Köse K.N., Ozbeyli D., Soluk-Tekkesin M., Kuru L. (2018). Evaluation of the effectiveness of esterified hyaluronic acid fibers on bone regeneration in rat calvarial defects. BioMed Res. Int..

[6] Li R., Guo W., Yang B., Guo L., Sheng L., Chen G., Tian W. (2011). Human treated dentin matrix as a natural scaffold for complete human dentin tissue regeneration. Biomaterials.

[7] Maureira M., Cuadra F., Cádiz M., Torres M., von Marttens A., Covarrubias C. (2021). Preparation and osteogenic properties of nanocomposite hydrogel beads loaded with nanometric bioactive glass particles. Biomed. Mater..

[8] Jayash S.N., Hashim N.M., Misran M., Ibrahim N., Al-Namnam N.M., Baharuddin N.A. (2021). Analysis on efficacy of chitosan-based gel on bone quality and quantity. Front. Mater..

[9] Jayash S.N., Cooper P.R., Shelton R.M., Kuehne S.A., Poologasundarampillai G. (2021). Novel chitosan-silica hybrid hydrogels for cell encapsulation and drug delivery. Int. J. Mol. Sci..

[10] Sultan N., Jayash S.N. (2023). Evaluation of osteogenic potential of demineralized dentin matrix hydrogel for bone formation. BMC Oral Health.

[11] Jing X., Xie B., Li X., Dai Y., Nie L., Li C. (2021). Peptide decorated demineralized dentin matrix with enhanced bioactivity, osteogenic differentiation via carboxy- methyl chitosan. Dent. Mater..

[12] Holiel A.A., Mahmoud E.M., Abdel-Fattah W.M., Kawana K.Y. (2021). Histological evaluation of the regenerative potential of a novel treated dentin matrix hydrogel in direct pulp capping. Clin. Oral Investig..

[13] Eshkol-Yogev I., Kaufman A., Haddad M., Zilberman M. (2022). Cell viability of novel composite hydrogels loaded with hydroxyapatite for oral and maxillofacial bone regeneration. Odontology.

[14] Kim B.J., Kim S.K., Lee J.H. (2021). Bone regeneration of demineralized dentin matrix with platelet-rich fibrin and recombinant human bone morphogenetic protein-2 on the bone defects in rabbit calvaria. Maxillofac. Plast. Reconstr. Surg..

[15] Gomes P.S., Fernandes M.H. (2011). Rodent models in bone-related research: The relevance of calvarial defects in the assessment of bone regeneration strategies. Lab. Anim..

[16] Takagi K., Urist M.R. (1982). The reaction of the dura to bone morphogenetic protein (BMP) in repair of skull defects. Ann. Surg..

[17] Shiu S.T., Lee W.F., Chen S.M., Hao L.T., Hung Y.T., Lai P.C., Feng S.W. (2021). Effect of different bone grafting materials and mesenchymal stem cells on bone regeneration: A micro-computed tomography and histomorphometric study in a rabbit calvarial defect model. Int. J. Mol. Sci..

[18] Ranganath S.K., Schlund M., Delattre J., Ferri J., Chai F. (2022). Bilateral double site (calvarial and mandibular) critical-size bone defect model in rabbits for evaluation of a craniofacial tissue engineering constructs. Mater. Today Bio.

[19] Naguib G.H., Abd El-Aziz G.S., Almehmadi A., Bayoumi A., Mira A.I., Hassan A.H., Hamed M.T. (2023). Evaluation of the time-dependent osteogenic activity of glycerol incorporated magnesium oxide nanoparticles in induced calvarial defects. Heliyon.

[20] Wadhwa P., Lee J.H., Zhao B.C., Cai H., Rim J.S., Jang H.S., Lee E.S. (2021). Microcomputed tomography and histological study of bone regeneration using tooth biomaterial with BMP-2 in rabbit calvarial defects. Scanning.

[21] Um I.W., Ku J.K., Lee B.K., Yun P.Y., Lee J.K., Nam J.H. (2019). Postulated release profile of recombinant human bone morphogenetic protein-2 (rhBMP-2) from demineralized dentin matrix. J. Korean Assoc. Oral Maxillofac. Surg..

[22] Nam J.W., Kim M.Y., Han S.J. (2016). Cranial bone regeneration according to different particle sizes and densities of demineralized dentin matrix in the rabbit model. Maxillofac. Plast. Reconstr. Surg..

[23] Xu J., Liu Y., Hsu S.H. (2019). Hydrogels Based on Schiff Base Linkages for Biomedical Applications. Molecules.

[24] Zheng A., Cao L., Liu Y., Wu J., Zeng D., Hu L., Jiang X. (2018). Biocompatible silk/calcium silicate/sodium alginate composite scaffolds for bone tissue engineering. Carbohydr. Polym..

[25] Venkatesan J., Bhatnagar I., Manivasagan P., Kang K.H., Kim S.K. (2015). Alginate composites for bone tissue engineering: A review. Int. J. Biol. Macromol..

[26] García-García P., Reyes R., Pérez-Herrero E., Arnau M.R., Évora C., Delgado A. (2020). Alginate-hydrogel versus alginate-solid system. Efficacy in bone regeneration in osteoporosis. Mater. Sci. Eng. C.

[27] Kamal M., Andersson L., Al-Asfour A., Bartella A.K., Gremse F., Rosenhain S., Gabato S., Hölzle F., Kessler P., Lethaus B. (2019). Bone regeneration in rabbit calvarial critical-sized defects filled with composite in situ formed xenogenic dentin and biphasic tricalcium phosphate/hyroxyapatite mixture. J. Biomed. Mater. Res. Part B Appl. Biomater..

[28] Mordenfeld A., Hallman M., Lindskog S. (2011). Tissue reactions to subperiosteal onlays of demineralized xenogenous dentin blocks in rats. Dent. Traumatol..

[29] Al-Asfour A., Andersson L., Kamal M., Joseph B. (2013). New bone formation around xenogenic dentin grafts to rabbit tibia marrow. Dent. Traumatol..

[30] Andersson L. (2010). Dentin xenografts to experimental bone defects in rabbit tibia are ankylosed and undergo osseous replacement. Dent. Traumatol..

[31] Andersson L., Ramzi A., Joseph B. (2009). Studies on dentin grafts to bone defects in rabbit tibia and mandible; development of an experimental model. Dent. Traumatol..

[32] Fricain J.C., Schlaubitz S., Le Visage C., Arnault I., Derkaoui S.M., Siadous R., Catros S., Lalande C., Bareille R., Renard M. (2013). A nano-hydroxyapatite–pullulan/dextran polysaccharide composite macroporous material for bone tissue engineering. Biomaterials.

[33] Fu S., Ni P., Wang B., Chu B., Peng J., Zheng L., Zhao X., Luo F., Wei Y., Qian Z. (2012). In vivo biocompatibility and osteogenesis of electrospun poly (ε-caprolactone)–poly (ethylene glycol)–poly (ε-caprolactone)/nano-hydroxyapatite composite scaffold. Biomaterials.

[34] Liu W., Wei Y., Zhang X., Xu M., Yang X., Deng X. (2013). Lower extent but similar rhythm of osteogenic behavior in hBMSCs cultured on nanofibrous scaffolds versus induced with osteogenic supplement. ACS Nano.

[35] Galle J., Bader A., Hepp P., Grill W., Fuchs B., Kas J.A., Krinner A., MarquaB B., Muller K., Schiller J. (2010). Mesenchymal stem cells in cartilage repair: State of the art and methods to monitor cell growth, differentiation and cartilage regeneration. Curr. Med. Chem..

[36] Du Z., Feng X., Cao G., She Z., Tan R., Aifantis K.E., Zhang R., Li X. (2021). The effect of carbon nanotubes on osteogenic functions of adipose-derived mesenchymal stem cells in vitro and bone formation in vivo compared with that of nano-hydroxyapatite and the possible mechanism. Bioact. Mater..

[37] Liu G., Xu G., Gao Z., Liu Z., Xu J., Wang J., Zhang C., Wang S. (2016). Demineralized Dentin Matrix Induces Odontoblastic Differentiation of Dental Pulp Stem Cells. Cells Tissues Organs.

